# Nanostructured Drug Delivery Systems for Targeting 5-α-Reductase Inhibitors to the Hair Follicle

**DOI:** 10.3390/pharmaceutics14020286

**Published:** 2022-01-26

**Authors:** Silvia Tampucci, Valentina Paganini, Susi Burgalassi, Patrizia Chetoni, Daniela Monti

**Affiliations:** Department of Pharmacy, University of Pisa, 56126 Pisa, Italy; valentina.paganini@phd.unipi.it (V.P.); susi.burgalassi@unipi.it (S.B.); patrizia.chetoni@unipi.it (P.C.); daniela.monti@unipi.it (D.M.)

**Keywords:** drug delivery, nanostructures, hair follicle, skin, androgenetic alopecia, finasteride, dutasteride

## Abstract

Androgenetic alopecia is a multifactorial condition characterized by noticeable hair loss, affecting both men and women and representing a debilitating and chronic disorder that considerably affects the quality of life. Available topical treatments based on minoxidil or finasteride require repeated applications and are associated with a certain number of adverse effects. The challenges associated with current treatments pave the way for the research of new therapeutic strategies, more precise and selective, and capable of providing long-term results. In this context, the present review examines the new proposed formulation strategies to deliver 5-α-reductase inhibitors in order to obtain a targeted drug delivery, for improving drug retention at the site of action in the hair follicle, contemporaneously reducing drug systemic absorption, which is the cause of important adverse effects. In particular, the research will be focused on the several aspects that influence the performance of nanostructured drug delivery systems in creating a depot in the hair follicles, such as particle size, surface charge, excipients, and combined application with external stimuli (infrared radiation, mechanical massage, ultrasounds application).

## 1. Introduction

Androgenetic alopecia, in males also referred to as male-pattern baldness, is the classic thinning of hair occurring in middle-aged men and increasing directly with ageing. Its incidence varies by race and is more commonly seen in white men than in Asians, American Indians and Africans [1,2,3]; women can also be affected, although less frequently and with a different pattern of hair loss [4,5,6]. 

Although it is known that common baldness in men has an autosomal dominant transmission while in women the phenotype is recessive, or even that baldness is genetically influenced, studies on this are still few, and the gene clearly responsible has yet to be identified, although genetics appear to be X-linked [7,8].

It is interesting to note that the testosterone hormone plays an important role in male androgenetic alopecia, regardless of genetic predisposition [9]. 

Testosterone, in the cells of the hair follicle, is converted into dihydrotestosterone (DHT) by the enzyme 5α-reductase, which exists as two isoenzymes: type I and type II. The latter, expressed in the hair follicles and other androgen-dependent tissues (i.e., the prostate gland), appears to be particularly important in male pattern baldness [1,10,11].

Androgen activity is mediated by binding to the human androgen receptor and the receptor–ligand complex regulates the expression of androgen-sensitive genes, acting as a transcription factor. The androgen receptor plays an important role in human development and is involved in the normal functioning of the reproductive system, the nervous system, and the immune system throughout the adult life. It is also involved in various diseases such as prostate cancer, androgen insensitivity syndrome and spinal and bulbar muscular dystrophy. Regarding baldness, it is the complex DHT-androgen receptor that acts as a transcription factor, regulating genes that control the hair cycle. 

**Figure 1:**
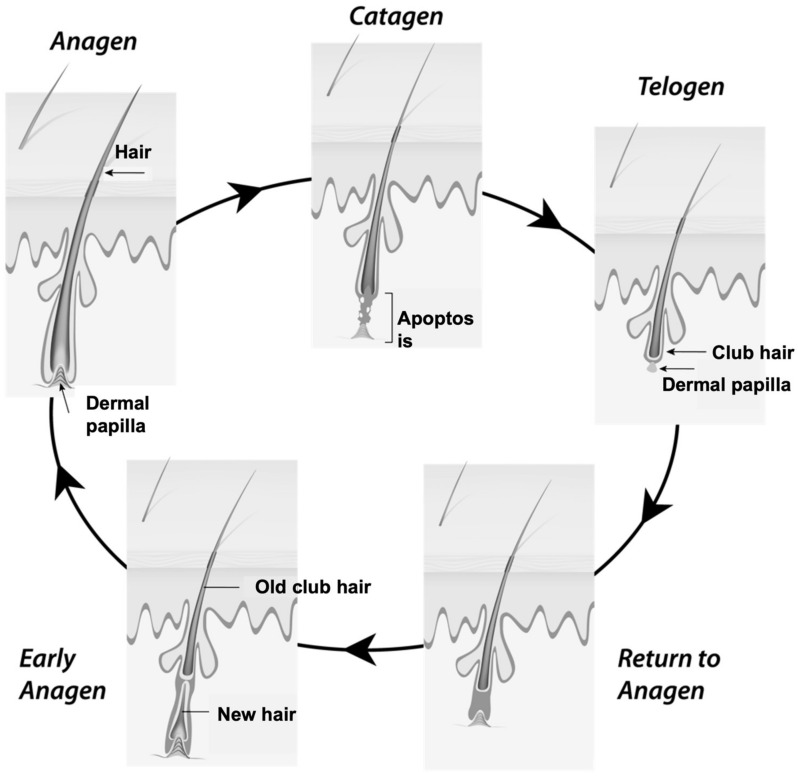
The normal hair cycle.

The normal hair cycle (Figure 1) consists of an active growth phase (anagen), which lasts from 2 to 6 years, followed by a short transition phase (catagen) of 1–2 weeks and subsequently by a resting phase (telogen), lasting about 3 months, during which the follicular sac, which contains the hair bulb, rises towards the epidermis. The hair then falls out, the anagen phase restart and a new hair is growing [1,4].

In the altered hair cycle typical of baldness, the phases remain the same, but the anagen phase is reduced, while the telogen resting phase lengthens with each new passage of the hair cycle, resulting in hair thinning and decreased hair length [1,4]. Finally, the anagen phase becomes particularly short that the new hair cannot reach the skin surface the period between the telogen phase of hair loss and the anagen phase of regrowth becomes longer, resulting in a reduction in the number of hairs present on the scalp. Moreover, these changes in the hair cycle are accompanied by a global follicular miniaturization that affects the papilla, the matrix, and the hair shaft [1,4,12]. 

For these reasons, androgenetic alopecia can be considered a debilitating and chronic condition that considerably affects the quality of life. In this context, the present research aims at investigating the drugs available on the market and evaluating the new formulation strategies to improve topical therapy. In particular, the proposed tools to obtain drug retention at the site of action in the hair follicle will be examined, in order to reduce drug systemic absorption, which is the cause of important adverse effects.

## 2. Current Treatments 

Currently, minoxidil and finasteride are employed for the treatment of androgenetic alopecia; they have different mechanisms of action and routes of administration, and they can be used both alone and in combination with each other.

Minoxidil is a powerful arterial vasodilator whose mechanism of action consists in opening the ATP-dependent calcium channels of the arterial smooth muscle cells which causes relaxation of the vascular smooth muscle.

The action of minoxidil in androgenetic alopecia therapy is not yet completely understood, even though the vasodilatory activity and the promotion of angiogenesis, as well as the ability to increase vascular endothelial growth factor (VEGF) [13] are considered to be possible mechanisms. At the level of the scalp, it leads to an increase in local circulation due to vasodilation with a consequent increase in the availability of oxygen and nutrients for the cells of the hair bulb [14].

Initially, minoxidil was born as a drug for systemic use for the treatment of hypertension, but in 1988 it was firstly authorized for use by FDA in the treatment of male androgenetic alopecia in a topical solution with the trade name Rogaine^®^, currently available. The side effects of topical administration have so far been limited to local skin irritation, pruritus, and reversible hypertrichosis. 

Finasteride is a competitive inhibitor of the 5α-reductase type II enzyme and prevents the conversion of testosterone into DHT. It has been approved for treatment of androgenetic alopecia in a daily dosage per os of 1 mg, which can decrease the levels of circulating DHT in the serum and scalp by 60%. However, the clinical response to finasteride therapy is highly variable and systemic use can lead to the onset of adverse effects such as sexual dysfunction, depression, and gynecomastia [13].

It is commercially available, as Proscar^®^ (5 mg tablet for the treatment of benign prostatic hypertrophy) and Propecia^®^ (1 mg tablet for the treatment of AGA). Finasteride has been reported to be effective at a concentration between 0.25% and 5% in topical solution [14]. Recently, the marketing in the European Union of a topical formulation containing finasteride in a concentration of 2.275 mg/mL under the name of Caretopic^®^ was authorized for the treatment of androgenetic alopecia. The topical treatment possesses a less severe side effect profile if compared to systemic therapy, being mild skin irritation at the application site the most frequent effect observed [15]. An important aspect to be pointed out is the risk of transfer to third parties for contact with the treated surface; therefore, attention should be paid during pregnancy for teratogenicity for male fetuses.

Table 1 summarizes the pharmaceutical forms on the market containing minoxidil and finasteride for the treatment of alopecia.

A drug currently of interest for the treatment of androgenetic alopecia is dutasteride, an inhibitor of the 5α-reductase enzyme that inhibits both isoforms of the enzyme by bocking or competing with NADPH, necessary for the enzyme to convert free testosterone into DHT. Dutasteride is three times more effective than finasteride in inhibiting isoform I and one hundred times more effective against isoform II, and this makes it significantly more powerful: a 0.5 mg dose of dutasteride leads to a reduction in circulating DHT 90%. Dutasteride is currently on the market as Avodart^®^ for the treatment of benign prostatic hypertrophy. Although its use for the treatment of androgenetic alopecia has not yet been approved, this drug appears to be very promising and worthy of consideration in this area [13].

## 3. Drug Delivery to the Hair Follicle

Most of the formulations marketed for this purpose are represented by solutions with a high percentage of propylene glycol and/or ethanol, excipients responsible in some cases of serious adverse effects following repeated applications (e.g., dry scalp, irritation, allergic contact dermatitis, burning, redness). To reduce cutaneous adverse effects and to improve therapeutic efficacy, the interest of the research has focused on the development of not irritating formulations that could deliver the active compound to the superficial layers of the skin and inside the hair follicle, to obtain a sustained drug release [16].

**Figure 2:**
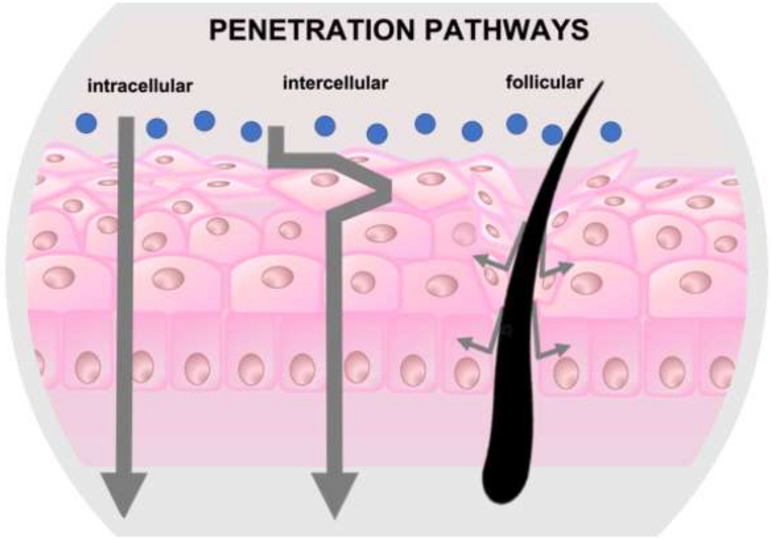
Potential routes for a molecule to cross the skin.

For topically administered substances, percutaneous absorption can occur via the intercellular, transcellular or through the skin appendages; the latter including the transport through the hair follicles and the ducts of the sebaceous and sweat glands (Figure 2).

The main barrier to skin permeation is represented by the stratum corneum, the outermost layer of the skin, which consists of cornified cells surrounded by lipids; the major route for skin penetration is certainly the intercellular route, although in recent years the penetration through the skin appendages (also called shunt route) has proved to be important in some specific cases. 

The shunt pathway has received renewed interest because it appears to be the main one for nanoparticles, liposomes and hydrophilic drugs and large polar steroid molecules that are not expected to pass quickly through the skin.

From the moment a topically applied substance enters the hair follicle, it can follow different routes. Small drugs can penetrate deeply through the follicular epithelium and reach the bloodstream. On the other hand, substances that are too large to penetrate, i.e., particles, are trapped inside the hair follicles and can be depleted only following slow processes such as hair growth and sebum secretion [17].

The design of formulations for drug delivery at the hair follicle level must take into account the complex anatomical structure and cyclic activity of the follicles, whose size and density vary according to the body area [18]. The openings of the hair follicles occupy almost 0.1% of the total skin surface and in regions such as the scalp and face they reach up to 10% [16].

In addition to anatomical characteristics, a large variety of factors influence drug deposition in the pilosebaceous unit, for example the type of formulation or the drug delivery system, humidity, temperature, characteristics of the molecule to be administered (i.e., size, polarity) and the penetration depth to be achieved [18]^.^

Vogt et al. [19] defined three important targets in the hair follicle:

- The infundibulum, which participates in the increase in surface area and interrupts the epidermal barrier down to the lower portion of the follicles, constituting an efficient reservoir for the permeation of substances;
- The sebaceous gland, which is related to the follicle through the sebaceous duct; and
- The area of the hair bulb, where the stem cells of the germinative matrix are found and appear as an ideal target for the therapy of skin diseases. The cells in the hair matrix may represent potential target sites for hair growth control [17].

In the case of androgenetic alopecia, a direct targeting of the area of the hair bulb is desirable, as the entire process of DHT formation from testosterone via 5-α-reductase and the subsequent binding of DHT with androgen receptors is settled in the dermal papilla cells [20]. 

### 3.1. Influence of the Particle Size

Many reports have evaluated the differences in follicular deposition after application of drugs in different vehicles, and in particular, nanostructured drug delivery systems are currently a subject of intense research interest, because of their tendency to be retained in the hair follicle [21]. These systems can aggregate and accumulate in the follicular ostium, then penetrating the follicular canal according to their size [16]. 

**Figure 3:**
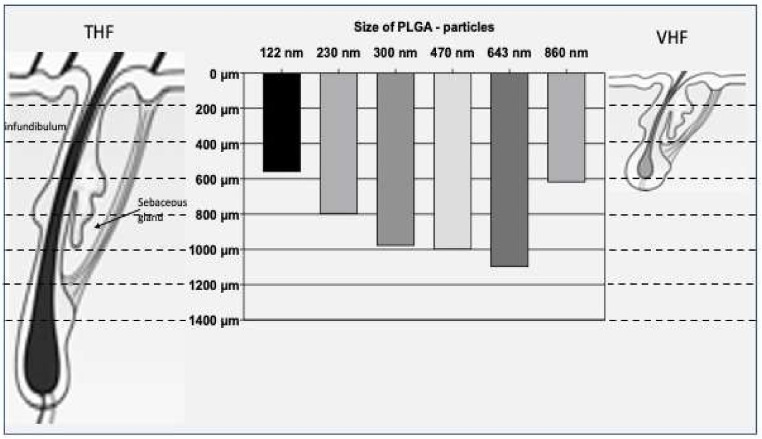
Penetration depths (µm) of different sizes of PLGA particles related to the target sites in terminal hair follicles (THF) and vellus hair follicles (VHF) (Reproduced from Blume-Peytavi et al. [18] which is licensed under a Creative Commons Attribution-(CC BY 4.0) International License http://creativecommons.org/licenses/by/4.0/).

The infundibulum, the sebaceous gland and the bulge region represent the relevant target structures within the hair follicle and can be selectively addressed in dependence of different particle sizes, as shown in Figure 3. 

Researchers have reported that the penetration of nanostructured drug delivery systems inside the follicular opening could be dependent on their diameter; therefore, nanoparticles can be retained in the hair shafts more than microparticles [17].

Generally, hair follicle targeting seems to be more efficient if the drug is encapsulated or attached to a nanocarrier, preferably around 400–700 nm in size. 

In cases where the infundibular region represents the target zone, the smallest dimensions around 122 nm and the much larger ones (860 nm) seems to be the most suitable, while intermediate dimensions around 230–300 nm tend to penetrate into the region of the sebaceous glands. On the other hand, particles with a size of 470–643 nm penetrate deeply into the hair follicle, reaching the bulge region [17].

It has been demonstrated that particles of 320 nm in size can be retained in the follicle for more than 10 days [18]. It is in fact hypothesized that particles with an average size of 300 nm can easily penetrate the hair follicles in reason of the cuticle thickness, which is about 530 nm in human hair and 320 nm in porcine hair [17,22]. Additionally, Ossadnik et al. [23] demonstrated that the cuticle thickness of the hair shaft amounts to approximately 600 nm.

Patzelt et al. [17] indicated that particles in the 400–700 nm range can penetrate deeply with respect to larger or smaller particles, probably due to the structure of the hair and the hair follicles. In addition, particle uptake or penetration depth seem to be independent of material properties. 

In the case of PLGA-particles, Patzelt et al. [17] reported that significantly higher penetration was achieved with increasing particle size (122 < 230 < 300 < 470 < 643 nm) with no further increase after a dimensional threshold. Furthermore, it was observed that particles belonging to neighboring size classes showed a statistically greater penetration depth for those with larger sizes. However, the 643 nm PLGA-particles showed the deepest penetration, and larger particles (860 nm) were able to penetrate significantly less deeply.

On the other hand, regarding silica particles, the same authors determined that the deepest penetration was achieved by particles with hydrodynamic diameter of 646 nm, while significantly lower penetration depths were shown by both smaller and larger particles (300 nm and 920–1000 nm, respectively). 

Comparison of PLGA and silica particles revealed comparable penetration depths for comparable particle sizes (300 and ≅640 nm), even though silica particles were able to penetrate more deeply than PLGA-particles of the same size.

Other nanoparticles such as polymeric nanoparticles have been proposed to be retained in hair follicles that can act as a long-term reservoir [24]. It has been reported that both the natural movement of the hair and the scaly structure of the hair cuticle can function as a gear pump capable of transporting appropriately sized particles into the follicle [25]. 

Several nano-sized drug delivery systems have been investigated, for example, penetration enhancer-containing vesicles able to promote drug deposition in the skin and nanoparticles (named “squarticles”) obtained from sebum-derived lipids that produced a 7-fold increase in the follicular uptake of minoxidil with respect to the control [26]. 

Lademann et al. [27] found that particles of 320 nm in size penetrated much more efficiently into the hair follicles than non-particulate substances. Indeed, a penetration depth of 1500 µm was reached by the particulate formulation, whereas the non-particulate formulation entered the hair follicles only to a depth of 400 µm. Toll et al. [28] produced biopsies for histological analysis of porcine ear skin to compare the penetration into the hair follicles of nanospheres in the diameter range 750 nm–6 µm. They observed that the smallest particles penetrated more efficiently into the hair follicles with respect to the larger ones [27].

Rancan et al. [29] also suggested that nanostructured systems possess a great potential for targeted drug delivery, by demonstrating that polylactic acid (PLA) nanoparticles with sizes of 228 nm and 365 nm penetrated human hair follicles, releasing loaded dyes into the surrounding tissues.

However, particles of different size have also been proposed to penetrate hair follicles, for example polystyrene particles 20 nm in size and iron-based particles [30].

Several studies have suggested that nanoparticles are able to deeply penetrate into hair follicles after topical application to human and animal skin, with some researchers suggesting that follicular delivery is more efficient with nanoparticles with diameters under 300 nm compared to larger nanoparticles [31,32,33,34].

Wu et al. [35] investigated polymeric nanoparticles with a mean diameter less than 50 nm after topical administration on porcine skin to assess both the drug release and the fate of the vehicle itself. 

The nanoparticles were not able to cross the stratum corneum, neither via the transcellular nor the intercellular route. Since the intercorneocyte space appears to be filled with multiple lipid bilayers, it seems unlikely that a particle 50 nm in diameter could be transported in the intercellular channels (100 nm); conversely, there seems to be a probability for particles to be seized in skin furrows and around follicles [35].

Another study showed that titanium oxide nanoparticles 20 nm in size penetrated the hair follicles up to 400 μm, but the presence of nanoparticles was not detected in vital tissues or in the sebaceous glands [36].

On the other hand, a study carried out in mice showed that the 40- and 200-nm-sized polystyrene nanoparticles retained in the hair spread into the surrounding tissue and reached the sentinel lymph nodes [37].

Moreover, Vogt et al. [34] found that Langerhans cells surrounding the hair follicles of human skin were able to internalize 40 nm nanoparticles, but not 750 nm and 1500 nm nanoparticles. They found that 40 nm nanoparticles penetrated along the follicular duct and entered the sebaceous duct to a depth of 225 µm, while in some cases they directly reached the perifollicular tissue. Conversely, 750 or 1500 nm particles were found neither below the upper infundibulum nor in the dermis [34].

Additionally, Schäfer et al. [38] demonstrated that polymeric microparticles with a diameter between 3 and 10 μm were able to selectively penetrate the follicular ducts, while particles larger than 10 μm remained on the skin surface and microspheres smaller than 3 μm were randomly distributed in the hair follicles and in the stratum corneum.

Based on the analyzed literature, summarized in Table 2, it can be concluded that nanostructured delivery systems represent a valuable strategy for drug delivery in the hair follicle and could be employed as a therapeutic option for topically applied drugs. So far, there is uncertainty about the optimal particle size necessary to produce a drug follicular penetration, but some pillars can be posed; 300–600 nm particles of different composition have been reported to be localized inside the hair follicle in the region below the sebaceous gland till the hair bulge, while particles outside this range (both above and below) seem to remain more superficially. 

Additionally, another dimensional range can be selected, represented by nanostructures with particle size below 100 nm, where data from the literature show more discrepancies. In this context, some attention must be paid to the delivery strategy if the perifollicular and living tissues are not the target sites, because occasionally smaller particles have been shown to permeate the follicular barrier.

Anyway, the drug size, the deformability and the shape of the particles could also represent variables in follicular targeting; therefore, further intensive research on these aspects is fully encouraged.

### 3.2. Influence of Excipients and External Stimuli

An important consideration in tuning up a drug delivery system is related to the abundance of sebum lipids in the hair follicle. The sebaceous gland with its secretion creates an environment rich in lipids. Indeed, the sebum is mainly made up of triacylglycerol, diacylglycerol, free fatty acids, squalene and cholesterol; therefore, the transport of drugs at this level is influenced by the interactions between sebum and drug and the choice of a suitable vehicle is fundamental. 

Dispersions of lipid nanoparticles, such as SLN or NLC, constituted of the same lipids, have also been proposed as a viable strategy for follicular targeting. Unfortunately, recent studies have highlighted some critical aspects of these nanocarriers compared to other topical formulations; in particular, non-specificity has been reported in the penetration at the level of the follicle, with simultaneous release on the skin surface of the encapsulated active ingredient [39]

The mechanism of penetration of the active compounds conveyed in a nanostructured form can be explained by the ratchet effect: the cuticular structure of the hair works as a ratchet that transports the particles deeper into the hair follicle, where they will remain for several days, forming a long-lasting reservoir of drug from which the drug is constantly released [40].

Moreover, in the dry state and at neutral pH, the skin surface is negatively charged because of many factors, such as the carbohydrate content, the presence of protein residues on the cell membranes, and active ions pumps; consequently, cationic solutes may be adsorbed on the skin surface [24]. 

Matos and colleagues [41] proposed the use of low molecular weight chitosan (75–85% of deacetylation), a cationic, biocompatible, naturally occurring polymer, commonly employed as permeation enhancer, to prepare a nanostructured system for targeting minoxidil to hair follicle in a non-irritating formulation. 

Additionally, positively charged liposomes showed enhanced localization at the level of the pilosebaceous unit, but the entrapped drug was poorly retained through the follicle length. Indeed, it is noteworthy that electrostatic interactions between the negatively charged skin surface and the positive nanocarrier can occur, increasing retention of the carrier in the upper skin layers and simultaneously reducing the permeation of actives in the deeper layers [39]. 

On the other hand, the skin can also present positive charges due to the presence of superficial lipids that cover the stratum corneum. Previous studies have shown that negatively charged carriers such as nanostructured lipid carriers (NLC) or solid lipid nanoparticles (SLN) are capable of interacting with lipids in the outermost skin layers, producing an occlusive effect. The negatively charged NLC and SLN, forming a film on the skin surface, may increase drug permeation across the stratum corneum and enhance drug retention in the skin [42]. 

Although drug delivery at the follicular target represents an important strategy for the topical administration of active ingredients at the cutaneous level, the development of new formulations to achieve this goal represents an important challenge. Fresta et al. [40] investigated a liquid crystal nanocarrier (LCN), able to self-assemble in a supramolecular structure to provide a site-specific delivery of minoxidil, used as model compound, in the hair follicles.

Another important aspect concerns rigidity and flexibility of the nanostructure, influencing permeation through the stratum corneum. Ultra-flexible nanoparticles are able to permeate through the stratum corneum and to deliver actives, as a function of the hydration gradient of the skin without producing any damages [29].

Studies have reported on biodegradable and nonbiodegradable nanoparticles, even though biodegradability is not so essential due to the probability that nanoparticles could be eliminated from the skin surface (e.g., due to hair growth, outflow of sebum, and epidermal turnover). Moreover, it is noteworthy that nanoparticles larger than 100 nm do not move from the follicles to living tissue [43].

However, it is important to highlight that, although it is now proven that nanocarriers are effective for drug targeting to the hair follicle, their formulation in appropriate vehicles is an important aspect to consider for the future development of a pharmaceutical product. Vehicles can be both hydrophilic and lipophilic and can present different viscosity, polarity, and many other parameters. A recent study of Pelikh et al. [40] investigated the influence of vehicle type on the penetration effectiveness of curcumin nanocrystals into hair follicles. Nanocrystals consist of 100% drug particles without any other matrix material, having high surface area and easy to be produced. Vehicles with different lipophilicity, viscosity and polarity were studied [44], and it was shown that vehicles with good skin moisturizing properties produced greater penetration of the active substance at the dermal level, simultaneously reducing follicular uptake. Therefore, it can be confirmed that the most influencing parameter is the ability of the vehicle to hydrate the stratum corneum. Stratum corneum hydration is linked with a reduced ratchet effect and could than result in a less efficient transport of the nanoparticles into the hair follicle. It has also been demonstrated that the excipients can strongly influence the penetration pathway. Addition of glycerol to nanocrystals hindered the hair follicle penetration and improved passive dermal penetration. Additionally, ethanol significantly enhanced the follicular penetration efficacy and reduced at the same time the drug passive penetration. Conversely, propylene glycol facilitated both penetration routes. Therefore, it can be concluded that adding different excipients to the formulation could result in the definition of a penetration pathway for the nanostructures (Table 3).

Based on the above considerations, the penetration of nanoparticles in the hair follicles can be described as a three-step process. In the first one, the particles must adhere loosely to the hair surface. In the second step, mainly influenced by the particle size, from the surface they must be positioned between the overlapping cells of the hair cuticle to take advantage of the ratchet mechanism. Finally, in the third step, the ratchet together with the pawl will then transport the particles into the hair follicle. 

In conclusion, it has been showed that the role of excipients may overlap with that of particle size. In addition, excipients can be considered to modify all three steps of the hair follicle penetration process. 

Therefore, in order to obtain a targeted penetration into the hair follicle, it is necessary not only to reach an optimal sizing of the nanoparticles, but also to select the appropriate excipients. Both aspects could be of interest in the development of the formulation of topical products [40].

Recent studies have also reported on the influence of external physical stimuli on the follicular targeting potential of different nanoparticles. The physical stimuli strategy is based on the observation that skin massage acts like a “gear pump” able to drive the transport of the nanoparticles into the hair follicle, together with the scaly structure of the hair cuticle. Similarly, it was observed that an effervescence reaction after administration of a solid powder formulation containing minoxidil can favor drug penetration [45]. The authors hypothesized that, as the particle diameter is larger than the follicular cast diameter (215–235 μm vs. 16–42 μm), the follicular targeting is the result of an active movement of the progressively smaller dissolved particles, rather than a passive deposition phenomenon [45].

As reported by Patzelt and Lademann [46], stimuli-responsive follicular targeting represents an interesting field to be explored. Indeed, gold nanotubes having an absorption band in the near IR can convert absorbed light energy into heat and can therefore act as a heat-controlled drug delivery system. 

Angelo et al. [25] also investigated NLC with a hydrodynamic diameter of 174 nm for follicle targeting. They found that the lipid carriers produced a high drug accumulation in the hair follicle, which is further enhanced by application of external physical stimuli, such as induced hyperthermia by infrared laser application, mechanical massage, and ultrasound application (frequency of 5 MHz and an intensity of 1.2 W/cm^2^) in pulsed mode with or without mechanical vibration, that can lead to a reduced drug dosage and less frequently drug application with greater safety and efficacy for patients.

## 4. Topical Formulations Containing 5-α-Reductase Inhibitors: State of the Art

In the preparation of formulations based on 5-α-reductase inhibitors, such as finasteride and dutasteride, one of the main problems encountered is due to the poor solubility of these therapeutic agents, which can result in low bioavailability [47,48]^.^ Therefore, the choice of suitable excipients and vehicles is crucial.

In recent years, interest has focused on the development of nanostructured systems that have great potential as a drug carrier for the treatment of dermatological diseases [49]. In fact, nanotechnologies allow the administration of drugs conveyed in systems of dimensions in the order of nanometers with consequent advantages such as increasing solubility, bioavailability, and efficacy of the therapeutic agent [50,51]. Among the nanostructured topical delivery systems of therapeutically active substances, a predominant role is covered by polymeric nanoparticles and liposomes [52,53].

Polymeric nanoparticles are colloidal carriers made up of a biodegradable, biocompatible, and non-toxic polymer, which can be natural or synthetic. Among the natural polymers, the most used for the preparation of nanoparticles for topical use is chitosan, which, being of a cationic nature, establishes strong interactions with the negatively charged skin surface. Furthermore, chitosan has antioxidant, anti-inflammatory and antimicrobial properties that make it particularly convenient in the treatment of dermatological pathologies [54]. On the other hand, poly lactic-*co*-glycolic acid (PLGA) and polylactic acid (PLA) are widely used as representative of synthetic polymers. Generally, the choice of the polymer considers the chemical-physical characteristics of the drug that must be encapsulated, attached, or dissolved in the nanoparticles; PLGA (75:25), for example, is used for the preparation of nanoparticulate systems comprising hydrophilic or hydrophobic drugs soluble in polar solvents [55]^.^

The dimensions of between 10 and 1000 nm allow the accumulation of nanoparticles at the level of the hair follicle and the skin surface making them extremely advantageous in the delivery of therapeutic agents at the topical level [50,56,57] 

Regarding 5-α-reductase inhibitors, several nanostructured drug delivery systems have been proposed in recent years, such as polymeric nanoparticles, liposomes, niosomes, ethosomes, liquid crystals and nanostructured lipid carriers [54,58,59,60]

Polymeric nanoparticles represent an efficient system for hair follicle drug targeting, with some polymers, such as PLA or PLGA, seeming more advantageous with respect to others. For example, Roque et al. [24] investigated polymeric nanoparticles based on PLGA (50/50) with a mean particle size of 300 nm that were able to encapsulate finasteride, showing a prolonged drug release with low skin permeation. Additionally, a recent study by Afiune et al. [61] proposed the use of iron oxide nanoparticles surface coated with lauric acid, also used as an inhibitor of types I and II of 5-α-reductase, for the delivery of dutasteride or finasteride. The authors found that the system was able to deliver the drug in the skin producing a higher retention with respect to the control. Furthermore, they found that the capability to accumulate the drug in the hair follicle was better when dutasteride was encapsulated with respect to finasteride, maybe due to disassembling of the iron oxide structure. Another recent study suggested that dutasteride formulated in a nanostructured lipid carrier coated with lauric acid-chitosan oligomer (chitosan MW < 3000 Da) represent an efficient tool for drug delivery in the skin depth, reducing both the drug permeation and cytotoxicity [54].

Liposomes are vesicular carriers in which an aqueous core is enclosed in one or more lipid bilayers mainly consisting of cholesterol and phospholipids [62]; the presence of one or more bilayer shells determines their size, as they can be classified either as small unilamellar vesicles (SUV 20–100 nm), large unilamellar vesicles (LUVs 100–1000 nm), giant unilamellar vesicles (GUV >1000 nm), oligolamellar (OLV 100–1000 nm), multilamellar vesicles (MLV > 500 nm) or multi-vesicular vesicles (MVVs >1000 nm) [63]. The lipid composition promotes liposomes’ absorption on the skin surface and their fusion with the lipids of the stratum corneum, with release of the drug into the tissue. Alternatively, they can be produced to obtain accumulation of the drug in the skin layers: liposomes with an average diameter of less than 50 nm showed accumulation in the deeper layers of the skin, as opposed to particles of larger diameter that are deposited in the superficial layers. The drug, depending on its physicochemical characteristics, can be entrapped in the aqueous space, or intercalated in the lipid bilayer of the liposome.

The use of liposomes allows improving the pharmacokinetics of the administered substance, also increasing its specificity and efficacy, and decreasing its toxicity [50,57].

In the literature, numerous vesicular systems (basically liposomes) for the delivery of drugs such as finasteride and dutasteride are described and some examples are summarized in Table 4.

Even though liposomes have been demonstrated to enhance drug delivery in the hair follicle for the treatment of androgenetic alopecia, their industrial application is limited due to chemical-physical instability and microbial contamination. Therefore, alternatives have been proposed by researchers, such as polymersomes, self-assembled spherical vesicles based on amphiphilic block copolymers. Caon et al. [69] investigated negatively charged polymersomes based on polystyrene (PS) and poly (acrylic acid) (PAA) block copolymers decorated with chitosan (average MW of 37,000 g mol^−1^ and degree of deacetylation around 85%) for the topical delivery of finasteride that were able to strongly interact with the skin, creating a drug depot in the epidermis and dermis.

In recent years, researchers’ interest has been aimed at investigating tools to control the drug release in response to some stimuli. Ushirobira et al. [70] proposed poly (ε-caprolactone)-lipid-core chitosan-coated (chitosan MW 50–190 kDa, 75% deacetylation) nanocapsules containing dutasteride for drug delivery to the hair follicle. The authors demonstrated both that nanocapsules can deliver the drug inside the follicle and that a massage as physical stimulation can enhance the drug retained.

Another proposed strategy is represented by formulating nanoemulsions decorated with biopolymers as polysaccharides and alginates [71]. 

Nanoemulsions are thermodynamically stable O/W or W/O dispersions with droplets’ dimensions on the order of nanometers stabilized with a low percentage of surfactants. They have an average diameter of dispersed phase droplets between 20 and 1000 nm. They are able to solubilize lipophilic drugs with a high loading capacity and the high contact surface with the stratum corneum allows an increase in permeation and the transport of drugs in the skin depth. Furthermore, the surfactants included in the composition are able to interact with the lipid structure of the stratum corneum, temporarily modifying the barrier, thus facilitating the passage of the drug.

Low-molecular-weight chitosan-decorated nanoemulsions containing finasteride showed low drug permeation and increased skin retention, even with droplet size of 800 nm, with respect to non-decorated nanoemulsions, maybe due to their positive charge and viscous nature [71]. 

Moreover, a patented nanoemulsion based on dutasteride and containing tocopheryl phosphates and tocotrienol phosphates as co-surfactants has been proposed [72].

Other formulative strategies to optimize the topical administration of drugs at the scalp level could be adopted to create a depot at the topical level and in recent years many patents have been developed.

In 2011, a formulation containing chitosan, a polymer capable of forming a film after the evaporation of volatile solvents allowing intimate contact between the therapeutic agent and the skin and increasing its hydration with consequent promotion of absorption, was patented [73].

Another formulation involves the use of lipids that form non-lamellar liquid crystals when they encounter biological fluids, obtaining a controlled drug delivery system [74]. A similar principle was exploited in another formulation consisting of a polymeric solution that, once intradermically administered, led to the formation of an in situ precipitate. The contact with water triggers the phase inversion of the polymer, which precipitates forming a polymeric gel. This mechanism allows obtaining a depot with controlled drug release [75].

Table 5 summarizes the characteristics and bibliographic references of the above-discussed patented formulations.

## 5. Future Perspectives 

Since androgenetic alopecia is specifically restricted to pilosebaceous units, improving the outcome of alopecia therapy might be possible by increasing drug distribution at the target site within the hair follicles. Follicular drug targeting will provide advantages in terms of reduction of the drug dose, while simultaneously minimizing drug systemic absorption.

Nanostructured drug delivery systems represent the most promising option for topical delivery, as they could improve drug-selective tissue distribution in the hair follicle region, minimizing side effects and reducing the required dose. Moreover, the use of nanotechnologies could improve drug solubility and contemporaneously increase the drug concentration at the target site by overcoming the skin barriers for penetration (stratum corneum and sebum). 

Indeed, the optimal dimension range to address the target site inside the hair follicle should be pursued in order to efficiently penetrate into the hair follicles and to reach the depth of the hair bulb. The particle size, the surface charge, and the effect of the combination of polymers and/or surfactants should be investigated in order to select the best formulation, capable also of interacting with sebum in order to facilitate drug partition in the hair shunt. Additionally external stimuli such as massage or ultrasound application could be considered as tools to improve drug retention inside the hair follicle.

## Figures and Tables

**Table 1:** Approved pharmaceutical forms and therapeutic indications for the treatment of androgenetic alopecia (AGA).

Pharmaceutical Form/Route	Therapeutic Indications	Regulatory Authority
	**Minoxidil**	
Solution, 2%; topical	AGA in male and female	AIFA, FDA
Solution 5%; topical	AGA in male	AIFA, FDA
Solution 20 mg/mL; topical	AGA in male and female	AIFA, FDA
Solution 50 mg/mL; topical	AGA in male	AIFA, FDA
Aerosol, foam, 5%; topical	AGA in male and female	AIFA, FDA
	**Finasteride**	
Tablets 1 mg; oral	AGA in male	AIFA, FDA
Sprayable solution 2.275 mg/mL; topical	AGA in male	AIFA

**Table 2:** Role of the formulation and of the particle size to reach the target region in the hair follicle.

Formulation	Particle Size	Target Region	Reference
Hydrogel suspension of PLGA particles containing fluoresceinamine	- 122 and 860 nm - 230–300 nm - 470–643 nm	- Infundibular region (≤600nm) - Sebaceous gland region (600–1000 nm) - Hair bulge (≥1000 nm)	[17]
Aqueous suspension of silica particles containing fluoresceinamine	- 300 nm and 920–1000 nm - 646 nm	- Infundibular and sebaceous gland region (≤1000 nm) - Hair bulge (≥ 1000 nm)	[7]
Colloidal suspension of polymeric nanoparticles with finasteride in water and Pluronic^®^ F68	316.5 nm	hair follicle	[24]
Nanostructured lipid carriers containing clobetasol	173.80 nm	hair follicle (with deepest depth obtained after 12 h)	[25]
Aqueous suspension of fluorescein labeled nanoparticles	320 nm	1500 μm depth	[27]
Fluoresbrite Yellow Green rigid Carboxylate Microspheres	- 750 nm - 6 μm	- homogenous distribution on the skin surface - tendency to aggregate in the follicular orifices and selective penetration route into the HF.	[28]
Suspension of polylactic acid (PLA) nanoparticles with fluorescent dye	228 nm and 365 nm	penetration in hair follicles and release of loaded dyes into the surrounding tissues.	[29]
Polystyrene and iron-based particles	20 nm	penetrate hair follicles	[30]
Podophyllotoxin-loaded solid lipid nanoparticles stabilized by poloxamer 188 (P-SLN) and soybean lecithin (T-SLN)	73.4 nm (P-SLN, negatively charged) and 123.1 nm (T-SLN)	- strong localization of POD within epidermis with P-SLN and skin penetration into skin along the hair follicle and epidermis	[31]
Aqueous nanoparticle suspension based on poly(ε-caprolactone)-block-poly(ethylene glycol) containing minoxidil	40 nm (S-NP) and 130-nm (L-NP)	hair follicles is the main pathway of this minoxidil-loaded nanoparticles	[32]
Polystyrene nanoparticles containing fluorescein 5-isothiocyanate	20 nm and 200 nm	preferential accumulation of FITC nanoparticles in the follicular openings	[33]
Nanoparticles suspension in PBS pH 7.4 with fluorescent probes	40 nm 750 or 1500 nm	- penetration along the follicular duct, reaching the sebaceous duct to a depth of 225 μm; reaching of the perifollicular tissue - neither below the upper infundibulum nor in the dermis	[34]
Polymeric nanoparticles	<50 nm	retention in skin furrows and around follicles	[35]
Titanium oxide nanoparticles	20 nm	- penetration in hair follicles up to 400 μm - no detection in vital tissues or sebaceous glands	[36]
Polystyrene nanoparticles	40 and 200 nm	- hair retention and subsequent spreading to surrounding tissues	[37]
Polymeric microparticles	< 3 μm 3 and 10 μm >10 μm	- randomly distributed in the hair follicles and in the stratum corneum. - selective penetration of the follicular ducts - remain on the skin surface	[38]

**Table 3:** Type and concentration role of some excipients on passive dermal penetration and hair follicle targeting; ↑: increase, ↔: do not affect, ↓: decrease. Reproduced from Pelikh et al. [40] which is licensed under a Creative Commons Attribution-(CC BY 4.0) International License (http://creativecommons.org/licenses/by/4.0/).

Type and Concentration of Excipient	Effect on Passive Dermal Diffusion	Effect on Hair Follicle Targeting
Glycerol 2%	↑	↔
Glycerol 5% *	↑↑↑↑	↓
Urea 5%	↑↑	↔
Urea 10%	↑	↓
Propylene glycol 5% **	↑↑↑↑	↑↑↑
EtOH 2% ***	↓	↑↑↑
Olive oil 2%	↔	↑

* Suggested for drug delivery via passive dermal penetration without depot effect. ** Suggested drug delivery via passive dermal penetration with simultaneous depot effect. *** Suggested drug delivery via hair follicles without passive dermal penetration.

**Table 4:** Examples of vesicular systems described in the literature for the topical treatment of AGA.

Formulation	Active Principle	Lipid Component	Particle Size and Surface Charge	Reference
Liposomes	Finasteride or minoxidil	cholesterol, cholesterol derivatives, sterols, PC ¹, PE ², PI ³, SPH ⁴, phosphatidic acid, mono-, di- and triglycerides derivatives galactolipids, mannolipids.	30–1000 nm n.a. *	[64]
Liposomes dispersed in aqueous gel (siliconic derivative ^a^ with occlusive effect)	Finasteride or dutasteride	PC ¹, PE ², PI ³, SPH ⁴, phosphatidic acid or sterols (cholesterol)	n.a. *	[65]
Liposomes associated with microbubbles	Finasteride, dutasteride or minoxidil	PC ¹, cholesterol and cationic phospholipids (e.g., DPPE ⁵)	n.a. *	[66]
Liposomes and Niosomes	Finasteride	DMPC ^6^, dicetyl phosphate, cholesterol, Brij 72 (polyoxyethylene 2 stearyl ether), Brij 76 (polyoxyethylene 10 stearyl ether), Brij 97 (polyoxyethylene 10 oleyl ether), Span 40 (sorbitan monopalmitate)	1.9–4.4 μm negatively charged	[58]
Multilamellar liposomes	Finasteride	PC ¹, cholesterol, dicetyl phosphate	15.4–24.1 μm neutral and negatively charged	[67]
Liposomes, hyalurosomes, glycerosomes and glycerol-hyalurosomes	Finasteride and baicalin	Lipoid^®^ S75, soybean lecithin	65–110 nm negatively charged	[68]

¹ Phosphatidilcholine ² Phosphatidil ethanolammine ³ Phosphatidilinositol ⁴ Sphingomyelin ⁵ Dipalmitoyl Phosphatidil ethanolammine, ^6^ Dimyristoyl phosphatidylcholine, ^a^ Silicon modified with hydrophylic groups, i.e., bis peg 18 methyl ether dimethyl siloxane. * n.a. data not available.

**Table 5:** Formulation strategies to create a depot at the topical level for the treatment of AGA.

Formulation	Active Principle	Composition	Strategy	Reference
Chitosan-based	Finasteride, dutasteride, others	Chitosan, chitosan derivatives, volatile solvent (i.e., ethanol)	Film-forming solution for topical application allowing long lasting scalp contact and continuous drug release	[73]
Lipidic solution	Finasteride o dutasteride in association with corticosteroids and/or FANS	Sorbitan esters with unsaturated fatty acids, phospholipids and retinyl palmitate, tocopherol acetate, cholesterol	Liquid crystal formation after formulation exposure to aqueous fluid after injection or topical application allowing a sustained drug release	[74]
Polymeric non aqueous solution	Finasteride, dutasteride, minoxidil	biocompatible and biodegradable polymers/water miscible organic solvent	Subcutaneous injection allow in situ gel depot formation and controlled and substained drug release	[75]
